# Opportunistic Colonoscopy Cancer Screening Pays off in Romania—A Single-Centre Study

**DOI:** 10.3390/diagnostics11122393

**Published:** 2021-12-19

**Authors:** Iulia Rațiu, Raluca Lupușoru, Prateek Vora, Alina Popescu, Ioan Sporea, Adrian Goldiș, Mirela Dănilă, Bogdan Miuțescu, Andreea Barbulescu, Madalina Hnatiuc, Razvan Diaconescu, Sorina Tăban, Fulger Lazar, Roxana Șirli

**Affiliations:** 1 Center for Advanced Research in Gastroenterology and Hepatology, Department of Internal Medicine II, Division of Gastroenterology and Hepatology, “Victor Babes” University of Medicine and Pharmacy, 300041 Timisoara, Romania; ratiu_iulia@yahoo.com (I.R.); alinamircea.popescu@gmail.com (A.P.); isporea@umft.ro (I.S.); goldisadi@yahoo.com (A.G.); mireladanila@gmail.com (M.D.); bmiutescu@yahoo.com (B.M.); barbulescu.andra91@gmail.com (A.B.); m.hnatiuc@yahoo.com (M.H.); roxanasirli@gmail.com (R.Ș.); 2 Center for Modeling Biological Systems and Data Analysis, Department of Functional Sciences, “Victor Babes” University of Medicine and Pharmacy, 300041 Timisoara, Romania; 3 Department of Gastroenterology, Saint Mary Hospital, 300203 Timisoara, Romania; drprateekvora@gmail.com; 4 Department of Surgery, Faculty of Medicine, “Vasile Goldiş” Western University of Arad, 310025 Arad, Romania; diaconescu_razvan@yahoo.com; 5 ANAPATMOL Research Center, Discipline of Morphopathology, Department of Microscopic Morphology, “Victor Babes” University of Medicine and Pharmacy, 300041 Timisoara, Romania; sorinataban@yahoo.com; 6 Department X, 2nd Surgical Clinic, Researching Future Chirurgie 2, “Victor Babeș” University of Medicine and Pharmacy Timișoara, 300041 Timisoara, Romania; lazarfulger@yahoo.com

**Keywords:** ADR, PDR, CRC, colonoscopy, opportunistic screening

## Abstract

Colorectal cancer (CRC) is the third most diagnosed cancer in men (after prostate and lung cancers) and in women (after breast and lung cancer). It is the second cause of cancer death in men (after lung cancer) and the third one in women (after breast and lung cancers). It is estimated that, in EU-27 countries in 2020, colorectal cancer accounted for 12.7% of all new cancer diagnoses and 12.4% of all deaths due to cancer. Our study aims to assess the opportunistic colorectal cancer screening by colonoscopy in a private hospital. A secondary objective of this study is to analyse the adenoma detection rate (ADR), polyp detection rate (PDR), and colorectal cancer (CRC) detection rate. We designed a retrospective single-centre study in the Gastroenterology Department of Saint Mary Hospital. The study population includes all individuals who performed colonoscopies in 2 years, January 2019–December 2020, addressed to our department by their family physician or came by themselves for a colonoscopy. One thousand seven hundred seventy-eight asymptomatic subjects underwent a colonoscopy for the first time. The mean age was 59.0 ± 10.9, 59.5% female. Eight hundred seventy-three polyps were found in 525 patients. Five hundred and twenty-five had at least one polyp, 185 patients had two polyps, 87 had three polyps, and 40 patients had more than three polyps. The PDR was 49.1%, ADR 39.0%, advanced adenomas in 7.9%, and carcinomas were found in 5.4% of patients. In a country without any colorectal cancer screening policy, polyps were found in almost half of the 1778 asymptomatic patients evaluated in a single private center, 39% of cases adenomas, and 5.4% colorectal cancer. Our study suggests starting screening colonoscopy at the age of 45. A poor bowel preparation significantly impacted the adenoma detection rate.

## 1. Introduction

Colorectal cancer (CRC) is the third most frequent cancer in men (after prostate and lung cancers) and in women (after breast and lung cancer) [1]. It is the second cause of cancer death in men (after lung cancer) and the third one in women (after breast and lung cancers). It is estimated that in European Cluster Collaboration Platform (EU-27) countries in 2020, colorectal cancer accounted for 12.7% of all new cancer diagnoses and 12.4% of all deaths due to cancer. This makes it the second most frequently occurring cancer (after breast cancer) and the second cause of cancer death (after lung cancer) in Europe. The overall colorectal cancer trends are increasing for incidence and decreasing for mortality, but there are national and regional exceptions and large variability among EU-27 countries. The five-year survival in colorectal cancer patients diagnosed in 2000–2007 is highest in Western Europe and lowest in some countries of Eastern Europe. National differences can, in part, be explained by different levels of healthcare expenditure and the resulting quality of screening, diagnosis, and treatment [2].

Romania has one of the highest colorectal cancer (CRC) incidence and mortality rates, and it is nearly double versus European ranges [3,4]. There is good evidence that a screening program reduces CRC incidence and mortality and is cost-effective compared to no screening, irrespective of the screening modality [5,6].

Current guidelines recommend the following intervals for screening:

Fecal immunochemical tests for hemoglobin (FIT) every 1 year; Colonoscopy every 10 years; Multitarget stool DNA test every 3 years; Flexible sigmoidoscopy every 5–10 years; Computed Tomography (CT) Colonography every 5 years; Colonic Capsule every 5 years [7,8,9].

Published papers on colorectal cancer screening by colonoscopy documented an adenoma and CRC detection rate of 14.9–37.5%, an advanced adenoma detection rate of 5–8.5%, an advanced neoplasia detection rate of 5–10.5%, and a complication rate between 0 and 0.3% [10,11,12].

There are two approaches to cancer screening. Organised screening occurs when all eligible individuals in a defined population are invited to be screened as part of an established program. By contrast, opportunistic screening occurs outside of an organised program and can be triggered only once an individual has been in contact with a health care practitioner [13].

Our study aims to assess the opportunistic colorectal cancer screening by colonoscopy in a private hospital. A secondary objective of this study is to analyse the adenoma detection rate (ADR), polyp detection rate (PDR), and colorectal cancer (CRC) detection rate.

## 2. Materials and Methods

### 2.1. Study Design

We performed a retrospective single-center study in a private referral hospital. The study population included all individuals who performed colonoscopies during 2 years, January 2019–December 2020, and who were addressed to our department by their family physician or who came by themselves for a colonoscopy.

### 2.2. Patients

The inclusion criteria were asymptomatic patients—average-risk individuals older than 40 years old that underwent a colonoscopy for the first time. The average risk was defined as without prior diagnosis of colorectal cancer, inflammatory bowel disease, familial polyposis syndromes, colonic adenomas, or colonic polyps; without a documented family history of colorectal cancer or prior colectomy; and no known history of prior colorectal cancer screening. The exclusion criteria included lower gastrointestinal hemorrhage, abdominal ballooning, transit disorders, or abdominal pain. We included all patients over 40 years old because we wanted to establish the optimal age for colorectal cancer screening. The patients between 40 and 50 years came by their own will for a colonoscopy; most of the patients were anxious and had a “fear of cancer”. The patients above 50 years were sent by a physician for colonoscopy colorectal cancer screening check, as recommended by the guidelines when patients come into their cabinet for other medical conditions such as liver steatosis, viral hepatitis, dyspepsia, and so on, even if they were asymptomatic for colorectal condition.

### 2.3. Colonoscopy Procedure

The patients were examined with a video colonoscope Fujinon EC600WL (Uetake, Kita-Ku, Japan) under sedation and analgesia with midazolam, propofol, and fentanyl performed by a senior anesthesiologist. Colonoscopies were performed in the morning and the afternoon by two fully trained endoscopists. Bowell preparation of the patients was either the full dose in the evening before or as a split regimen with a half dose in the morning, and we recorded the bowel cleanliness according to Boston Bowel Preparation Scale [14].

Every lesion was noted. If multiple lesions were found, we recorded them all. Adenomas were classified according to the WHO criteria: tubular, villous, and tubular–villous. Dysplasia was graded by Vienna classification [15,16]. Advanced adenoma was defined as a polyp in the colon or rectum with one of the following features: ≥ 1 cm as documented by the endoscopist, with villous architecture on histology or with high-grade dysplasia [17]. All identified polyps were resected during colonoscopy and examined by a senior pathologist.

As it was a retrospective study, according to Romanian legislation, ethical approval was not required. All patients signed written informed consent for the procedure and possible future studies. The study was conducted according to the Declaration of Helsinki.

### 2.4. Statistical Analysis

Data analysis was performed using MedCalc software v19.3. (Ostend, Belgium). Categorical variable comparisons were performed using the chi-square or Fisher exact test, and continuous variables were evaluated with the Student’s t-test or Mann–Whitney test. A *p*-value < 0.05 was used for significance. Factors associated with ADR were assessed using logistic regression analysis. For the evaluation of the performance in ADR, receiver operating characteristic (ROC) analysis was used. Optimal cut-off values were taken from corresponding receiver operating characteristic curves.

## 3. Results

### 3.1. Baseline Characteristics

One thousand seven hundred seventy-eight average-risk subjects underwent colonoscopy for the first time. The mean age was 59.0 ± 10.9, 59.5% female. The mean Boston score was 6.8 ± 1.3. Cecal intubation was photo-documented in 99.5% of cases (Table 1). No severe complication was noted after the colonoscopy.

873 polyps were found in 525 patients. Five hundred and twenty-five had at least one polyp, 185 patients had two polyps, 87 had three polyps, and 40 patients had more than three polyps. The polyp detection rate (PDR) was 49.1%, adenoma detection rate (ADR) 39.0%, advanced adenomas in 7.9%, and carcinomas were found in 5.4% of patients (Table 2).

#### Comparison between Age Groups and Gender

Of the 422 patients with adenomas, 50.9% (215/422) had more than one adenoma. Adenoma’s location was predominantly on sigmoid colon 30.4% (211/695), followed by rectum 22.4% (156/695), descendent colon 16.8% (117/695), transverse colon 11.9% (83/695), ascending colon 11.7% (81/695), cecum 4.3% (30/695) and rectosigmoid junction 2.5% (17/695). A total of 20.4% (142/695) patients had adenomas with high-grade dysplasia. Significantly more adenomas were found in men and women (31.4% vs. 18.3%, *p* < 0.0001). Regarding bowel preparation, in those that had good/excellent preparation, ADR was higher than in the group with poor preparation, 40.8% vs. 18.1%, *p* < 0.0001.

The lowest detection rate of advanced adenoma was found in the age group 50–75 years and the highest in the group above 75 years. The highest CRC detection rate was found in the age group above 75 years (Table 3). However, we divided the group under 50 years into two other groups, 40–44 years and 45–49 years. We had almost 6% (7/129) in the 45–49-years-old batch and 1.3% (3/233) in the 40–44 age group in totally asymptomatic individuals who do not fit into the screening population according to the guidelines. Regarding advanced adenoma, 5.1% (12/233) were found in 40–44 years and 11.6% (15/129) in 45–49 years, higher than in 50–75 years (5.9%), *p* < 0.001.

### 3.2. Factors Associated with ADR

**Figure 1:**
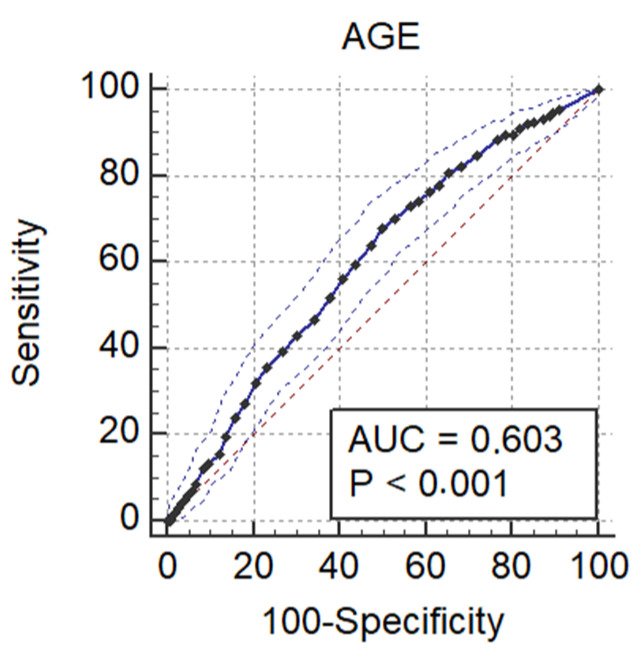
ROC analysis for age in detecting ADR.

We conducted a univariate logistic regression analysis that showed an association between ADR and age (*p* = 0.002) and bowel preparation (*p* = 0.01). Since age and bowel preparation were associated with ADR, we performed ROC analysis to assess their performance of age to predict adenoma detection. The optimal cut-off value for age was 45 years old, AUCs = 0.60, 95% CI (0.57–0.62), *p* < 0.001, Se = 91.9%, Sp = 49.8% (Figure 1), while for bowel preparation the cut-off was >6 pct, AUCs = 0.65, *p* = 0.01, Se = 84.1%.

## 4. Discussion

Screening for colorectal cancer applies to all middle-aged persons (45/50–75 y) at average risk for colorectal cancer (i.e., no prior CRC, adenomatous polyps, or inflammatory bowel disease; no prior diagnosis or family history of genetic predisposition to CRC, such as Lynch syndrome).

A single colonoscopy may be the most effective screening modality in preventing colorectal cancer incidence during 15 years follow-up. Colonoscopy screening is also associated with the highest risk for complications, but overall complication risks are low for colorectal screening [18].

In Romania, a national screening program is not yet available. Thus, patients often seek medical advice in the advanced stages of the disease. Due to this fact, we are practicing a so-called “opportunistic screening”, which includes asymptomatic patients—individuals who undergo screening either by their own will, patients mostly with anxiety, or as indicated by a physician seen for an unrelated colorectal condition (such as liver steatosis, dyspepsia, etc.) who were over 50 years. This is not a new concept; a few papers were published in Romania considering the lack of an organised screening program, but all came from large public hospitals [19,20]. Opportunistic colonoscopy is the most prevalent strategy even in the United States [21].

More than reported in other studies, we studied a large cohort (1778 patients) with a high cecal intubation rate of 99.5% [22]. PDR and ADR were 49.1% and 39%, respectively, similar to those described in the literature [23,24] and 5.4% for CRC. 

Most studies include patients at the age of 50–75 years for colorectal screening [25]. American College of Gastroenterology (ACG) 2021 guidelines recommend colorectal cancer screening in average-risk individuals of age 50 to 75 years and suggest screening in average-risk individuals of age 45 to 49 years because there are multiple challenges to initiating screening at age 45 years. One potential consideration is that if patients are screened by colonoscopy, they most likely will undergo more colonoscopies over their lifetimes than if screening begins at age 50 years or later [26]. European guidelines similarly recommend screening from the age of 50 years [27]. It is worth considering the additional benefits that early screening would yield for adults beyond 45–49 years of age. Lowering the starting age is likely to favorably impact the incidence and incidence-based mortality of the 50–54 age group, whose incidence and mortality are increasing.

Our study included patients under the age of 50 years old. In this particular group, the ADR CDR and advanced adenoma were higher than expected (33.9, 5.8, and 6.4%, respectively). After the ROC analysis to assess the performance of age in predicting ADR, we found the optimal cut-off value at 45 years. Since we do not have a screening program provided by a National Insurance Company, we cannot assess the cost-effectiveness of starting at a younger age, and we highly suggest starting the opportunistic screening by colonoscopy at the age of 45.

We also included patients over 75 years of age without significant comorbidities according to the recommendations of the AGA guidelines, which recommends personalised screening of patients in the age group of 75–85 years [28]. ADR was 34.2%, all adenoma were advanced, CCR was 17.1%, as expected, the highest rate in the study group.

The quality of bowel preparation assessed by the Boston Preparation Scale was higher than in other studies [29,30], probably because these patients performed the colonoscopy at their own will and expense. We had an ADR rate of 18.1% (25/138) in patients with poor bowel preparation, significantly lower than in the group with good to excellent bowel preparation −40.8% (670/1640) *p* < 0.0001. This finding suggests that the interval between screening colonoscopies in patients with poor preparation should be shorter.

Even though the study was conducted in a private gastroenterology center, it includes a large cohort of patients with a high cecal intubation rate, without any severe complications related to the procedure or anesthesia. Our study aims to draw attention to the urgent need in our country for an organised screening program and in the short term. However, we cannot draw a firm conclusion from a single-center study, and we suggest starting colorectal screening at the age of 45.

## 5. Conclusions

In a country without any colorectal cancer screening policy, polyps were found in almost half of the 1778 asymptomatic patients evaluated in a single private center, 39% of cases adenomas, and 5.4% colorectal cancer. Our study suggests starting screening colonoscopy at the age of 45. A poor bowel preparation significantly reduces the adenoma detection rate.

## Figures and Tables

**Table 1:** Patient’s characteristics.

Parameter	*N* (%), Mean ± SD
Age (years)	59.0 ± 10.9
Gender (female)	1050 (59.5%)
Boston score	6.8 ± 1.3
Good to excellent preparation (≥6)	1640 (92.2%)
Unsatisfactory to poor preparation (<6)	138 (7.7%)
Caecal intubation	1770 (99.5%)

*N* = number of patients; SD = standard deviation.

**Table 2:** Detection rates at colonoscopy.

Parameter	*N* (%)
Polyps	873 (49.1%)
Hyperplastic polyps	156 (8.7%)
Serrated polyps	22 (1.2%)
Adenomas	695 (39.0%)
Villous adenoma	21 (3.1%)
Tubular adenoma	378 (54.3%)
Tubular-villous adenoma	296 (42.5%)
Advanced adenomas	142 (7.9%)
Invasive carcinomas	96 (5.4%)
High grade dysplasia	246 (13.8%)

*N* = number of patients.

**Table 3:** Colonoscopy screening findings.

	Overall	<50 Years	50–75 Years	>75 Years	*p*-Value
Number	1.778	362	1305	111	-
Age (years)	59.0 ± 10.9	43.5 ± 1.6	61.6 ± 7.0	79.5 ± 2.9	-
Female	1050 (59.0%)	202 (55.8%)	788 (60.3%)	60 (54.0%)	0.17
Adenoma					
Number	695 (39.0%)	105 (29.0%)	555 (42.5%)	38 (34.2%)	0.01
Right size	501 (28.1%)	55 (15.1%)	374 (28.6%)	45 (32.1%)	0.001
Advanced	142 (7.9%)	27 (7.2%)	77 (5.9%)	38 (34.2%)	<0.0001
High dysplasia	246 (13.8%)	17 (4.6%)	241 (16.3%)	15 (13.5%)	<0.0001
Colorectal cancer	96 (5.4%)	10 (2.7%)	67 (5.1%)	19 (17.1%)	0.005

## Data Availability

The data presented in this study are available on requesr from the corresponding author. The data are not publicly available due to privacy reasons.
